# A closer look into DNase I hypersensitivity

**DOI:** 10.1186/1756-8935-6-S1-P25

**Published:** 2013-03-18

**Authors:** Housheng Hansen He, Clifford A Meyer, Henry Long, X Shirley Liu, Myles Brown

**Affiliations:** 1 Department of Biostatistics and Computational Biology, Dana-Farber Cancer Institute and Harvard School of Public Health, Boston, MA 02115, USA; 2 Department of Medical Oncology, Dana-Farber Cancer Institute and Harvard Medical School, Boston, MA 02115, USA; 3 Center for Functional Cancer Epigenetics, Dana-Farber Cancer Institute, Boston, MA 02215, USA

## 

DNase I hypersensitivity (DHS) combined with next generation sequencing (DNase-seq) is an efficient way of observing, in a single experiment, the genome-wide chromatin effects associated with the binding of multiple transcription factors. Using quantitative contrasts of DHS before and after estrogen and androgen stimulation in breast and prostate cancer cell lines, we have shown that differential DHS can accurately predict hormone induced transcription factor binding. Despite its effectiveness, the DHS assay can vary significantly depending on the experimental parameters. To increase the robustness of this assay, we have systematically evaluated two major parameters, digestion level and fragment size. We found that while there is a broad range of suitable digestion level, over-digestion dramatically decreases the efficiency of detecting DHS regions. More interestingly, we found that different fragment sizes capture distinct chromatin elements, and thus represent different chromatin structures. We were able to classify different combinations of estrogen receptor coregulators that resulted in different local chromatin structures.

